# Pre-stimulation of precision-cut bovine udder slices with zymosan before LPS exposure indicates aspects of trained immunity especially in the absence of FCS

**DOI:** 10.1177/17534259251360484

**Published:** 2025-08-21

**Authors:** Viviane Filor, Joanna Myslinska, Amitis Saliani, Jesmond Dalli, Peter Olinga, Wolfgang Bäumer, Dirk Werling

**Affiliations:** 1 School of Veterinary Medicine, Institute of Pharmacology and Toxicology, Freie Universität Berlin, Berlin, Germany; 2 Centre for Vaccinology and Regenerative Medicine, Department of Pathobiology and Population Sciences, Royal Veterinary College, North Mymms, UK; 3 William Harvey Research Institute, Barts and The London School of Medicine and Dentistry, 4617 Queen Mary University of London, London, UK; 4 Centre for Inflammation and Therapeutic Innovation, 4617 Queen Mary University of London, London, UK; 5 Groningen Research Institute of Pharmacy, Department of Pharmaceutical Technology and Biopharmacy, 3647 University of Groningen, Groningen, The Netherlands

**Keywords:** Precision cut bovine udder slices, trained immunity, lipidomics, bovine mastitis, immune response, specialised pro-resolving mediators

## Abstract

Mastitis in cattle poses a significant health challenge and results in substantial economic losses for the dairy industry. This study aimed to extend the existing precision-cut bovine udder slices (PCBUS) model as an *in vitro* model to explore the potential of inducing trained immunity in the udder with the goal to use the resulting knowledge for potential new treatment strategies. Interestingly, incubation of PCBUS with 10% fetal calf serum (FCS), but no 2% or FCS-free, negatively affected the production of some of the chemokines/cytokines analysed. When trained immunity was induced by zymosan, followed by stimulation with *E. coli*-derived lipopolysaccharide (LPS), production of interleukin (IL)-1β, IL-6, tumor necrosis factor α and interferon (IFNγ) was downregulated while production of IL-17A and pro-resolving lipid mediators (leukotrienes and prostaglandins) was upregulated. While the current experimental setup did not definitively confirm the induction of trained immunity for all parameters analysed in PCBUS, it validated the utility of PCBUS as a robust *in vitro* model for studying bovine udder inflammation. This model offers a promising platform for developing innovative mastitis treatments, particularly given the growing concern over antimicrobial resistance, as well as offering alternatives to the use of live animals in experimental studies in line with the 3Rs principles. It also provides a valuable tool for advancing our understanding of immune responses in the bovine udder. By adapting the precision-cut tissue slice technique to bovine udders, this model enables extensive research into new therapeutic approaches and supports basic research efforts to characterise complex pathophysiological processes associated with mastitis. Furthermore, our data highlight the potential limitations of FCS in *in vitro* studies. Our data should not only stimulate the discussion about FCS in homologues or heterologues species, but should also be kept in mind regarding the need for foetal calves to generate FCS in line with the 3Rs guideline.

## Introduction

Mastitis, an inflammation of the mammary gland, is the third most common health problems in dairy herds, affecting animal health and dairy economics worldwide.^1^ Mastitis is often caused by different strains of bacteria, and the literature indicates that more than 130 microorganisms have been implicated in the aetiology of mastitis, with the most common being *Staphylococcus aureus*, *Escherichia coli* or *Streptococcus uberis*, making treatment very complex.^2,3^ As bacterial infections are the main cause of mastitis in cattle, antibiotics have been the mainstay of treatment for decades. Astonishingly, approximately 80% of antibiotics used in the dairy industry are for the control and treatment of mastitis.^4–6^ For some pathogens, antimicrobial therapy has been shown to be very ineffective. In addition, the use of antibiotics is becoming increasingly difficult to justify in terms of bacterial resistance and consumer health.^7–9^ Despite considerable research efforts in recent decades, little is known about the immunological orchestra that plays a crucial role in pathogen eradication and tissue regeneration. As new developments are needed to overcome the lack of satisfactory mastitis therapy or good prevention strategies, new promising approaches need to be closely investigated.

Many studies have explored the idea of mastitis vaccination, but the low efficacy and slow progress of available commercial vaccines are partly due to a lack of knowledge of how vaccines work in the mammary gland and the exact mechanisms of protection against infection.^10–12^ This approach allows the hypothesis of innate immune memory, also known as trained immunity to be pursued. In certain mammalian vaccination models, protection against reinfection has been shown to occur independently of T and B lymphocytes.^13,14^ These observations have led to a concept in immunology known as “innate immune memory” or “trained immunity”.^15^ Innate immune memory differs from adaptive memory in many ways, including the absence of gene rearrangements, the involvement of epigenetic reprogramming, the type of cells involved, and the receptors involved in pathogen or antigen recognition.^16,17^ One of the known inducers of trained immunity is zymosan, a yeast cell wall derivative that is recognised by Dectin-1 and toll-like receptor (TLR)-2. It is known to contribute to the inflammatory response and cytokine production, and has been shown to induce an enhanced response to endotoxin challenge.^18–20^

Furthermore, the current literature supports the concept that polyunsaturated fatty acids (PUFA) can influence the biosynthesis of lipid mediators, effectively altering the functional capacity of cells involved in immune and inflammatory responses.^21,22^ Evidence has emerged indicating that resolution of acute inflammation is an active process with the biosynthesis of specialised pro-resolving mediators (SPM). Specialised pro-resolving mediators are a group of bioactive lipid mediators derived from omega-3 fatty acids that play a critical role in the resolution of inflammation. SPMs include resolvins, protectins, maresins and lipoxins.^23–25^ These molecules not only have anti-inflammatory effects, but also promote tissue repair and homeostasis after inflammation or infection. Their importance in the field of infection is increasingly being explored, as they have the potential to complement conventional antimicrobial therapy and thus combat the development of antibiotic resistance.^26–28^ SPMs are synthesised by the enzymatic oxidation of omega-3 fatty acids such as eicosapentaenoic acid (EPA) and docosahexaenoic acid (DHA). These mediators interact with specific G protein-coupled receptors (e.g., GPR32, GPR18, ALX) on immune and epithelial cells and modulate their function.^29,30^ SPMs promote the phagocytosis of pathogens by macrophages, inhibit the migration of neutrophils to the site of infection and support the clearance of dead cells and cellular debris. SPMs such as resolvins and maresins have been shown to reduce bacterial burden in several infection models by increasing the phagocytic activity of immune cells and inhibiting the release of pro-inflammatory cytokines.^31,32^

All these approaches are worth deeper investigation, especially in the target species. Precision-cut tissue slices (PCTS) allow the study of specific tissues or organs in an environment that closely resembles their natural physiological conditions.^33^ By using PCTS, researchers can investigate the local immune response and cellular interactions occurring within different infectious settings^33–35^ and are also used to investigate new therapeutic options and the metabolism of drugs.^36–38^ Therefore, it seems very promising to use this technique on the bovine udder to study the immune response in bovine mastitis in a physiological cellular setting.^39,40^ In these studies, we established the method for generating precision cut bovine udder slices (PCBUS) and showed that investigation of infections with mastitis pathogens is artificially possible and that the tissue responds to the infection.

In the first step of the current study, we expanded lthe already established system^39,40^ to PCBUS generated by the Krumdiek Tissue slicer. Initially, we investigated different culture conditions to see whether PCBUS can be cultured without the addition of FCS. It is not only the use of animals in science that raises ethical and scientific questions, but also the use of animal products. In addition to the ethical issues, the use of animal products can lead to contaminations that make research results unreliable. Since then, awareness of animal welfare in research has increased.^41,42^ We also investigated the cytokine profile after LPS stimulation to learn more about the orchestration of the immune system in the udder with or without FCS supplementation. After that, we decided to have a closer look to the idea of trained immunity within bovine udder tissue. Therefore, we pre-incubated the PCBUS with zymosan and stimulated them with LPS. The “high-throughput” potential of PCBUS, in conjunction with the concept of trained immunity in the udder will aid the discovery/testing of new approaches for the treatment of mastitis remains to be seen, but they provide at least screening abilities for these approaches.

## Materials and methods

### Precision-cut bovine udder slicing and *in vitro* culture

Precision-cut bovine udder slices (PCBUS) were obtained by using udders derived from Holstein-Frisian dairy cows humanly dispatched at a commercial abbatoir for non-mastitis related issues (Ethical approval URN 2024 2312-2). Subsequently, a piece of approximately 10 × 10 × 5 cm was cut out of the middle region of the bovine udder, immediately transferred into University of Wisconsin cold storage (UW)-solution and transported to the laboratory. Cutting the tissue into slices of roughly 1 cm, it was possible to punch out tissue cores with a diameter of 8 mm. To stabilise the tissue in the tissue core holder, the tissue was embedded in 3% agarose (w/v in 0.9% NaCl). PCBUS were prepared with a Krumdieck tissue slicer with a thickness of 250–350 µm. The slicer was filled with ice-cold phosphate-buffered saline (PBS), adjusted to a pH of 7.4. Promptly, slices were placed in a 24-well plate in 1 ml of pre-warmed and pre-oxygenated medium. Incubating slices was done in Roswell Park Memorial Insititute Medium (RPMI)-1640 medium supplemented with 1% penicillin-streptomycin, 1% fungizone and different concentrations of FCS (0%, 2% or 10%) at 37°C in presence of 5% CO_2_.

### Viability assay and morphological analysis

Slices were incubated in different concentration of fetal calf serum (FCS (Sigma-Aldrich, Cat No.: 12103C) serum free, 2% FCS and 10% FCS) as well as incubated with 1 µg/ml LPS from *E. coli* (O127:B8, Sigma) for 24 h. To assess cell viability, PCBUS were examined up to 24 h using AlamarBlue (PrestoBlue Assay (Thermo Fisher Scientific, UK)). PrestoBlue operates by converting resazurin (non-fluorescent) to resorufin (red fluorescent), with fluorescence intensity proportional to cellular viability and proliferation,^43^ 100 μL of PrestoBlue was added to a well containing a single PCLS in 900 μL of tissue culture medium and incubated for 1 h at 37°C, protected from direct light. The fluorescence intensity values were obtained using the Tecan Infinite 200 PRO Plate reader (Tecan Instruments) at 530/590 nm excitation/emission wavelengths. To generate negative viability control, three slices per each condition (per each medium compositon) were incubated in 100 μL of Triton-X 100 (Sigma-Aldrich, UK) for 30 min at room temperature (RT). Then, using a clean spatula, PCLS were submerged for 5 min in sterile PBS at 4°C, with PBS change performed thrice in total, to wash off the excess of Trion-X 100. Finally, slices were transferred to 900 μL of tissue culture medium for each of the treatments. To evaluate cell integrity of the PCBUS, HE-staining was performed. After the transfection, slices were fixed in 4% formalin at 4°C for 24 h. Afterwards, slices were dehydrated in ethanol with increasing concentrations. Slices were subsequently cleared in xylene. Thereafter, slices were horizontally embedded in paraffin and sectioned. Prior to staining with hematoxylin and eosin (H&E), sections were deparaffinized and rehydrated. Slides were scanned using an AxioScan Z1 slidescanner (Serial number: 4647000114 C) with Zen Blue (Version 3.1.) software package. The microscopic appearance of slices was assessed by evaluating the cytoplasm and the shape/staining of nuclei.

### Treatment of PCBUS

**Figure 1.**
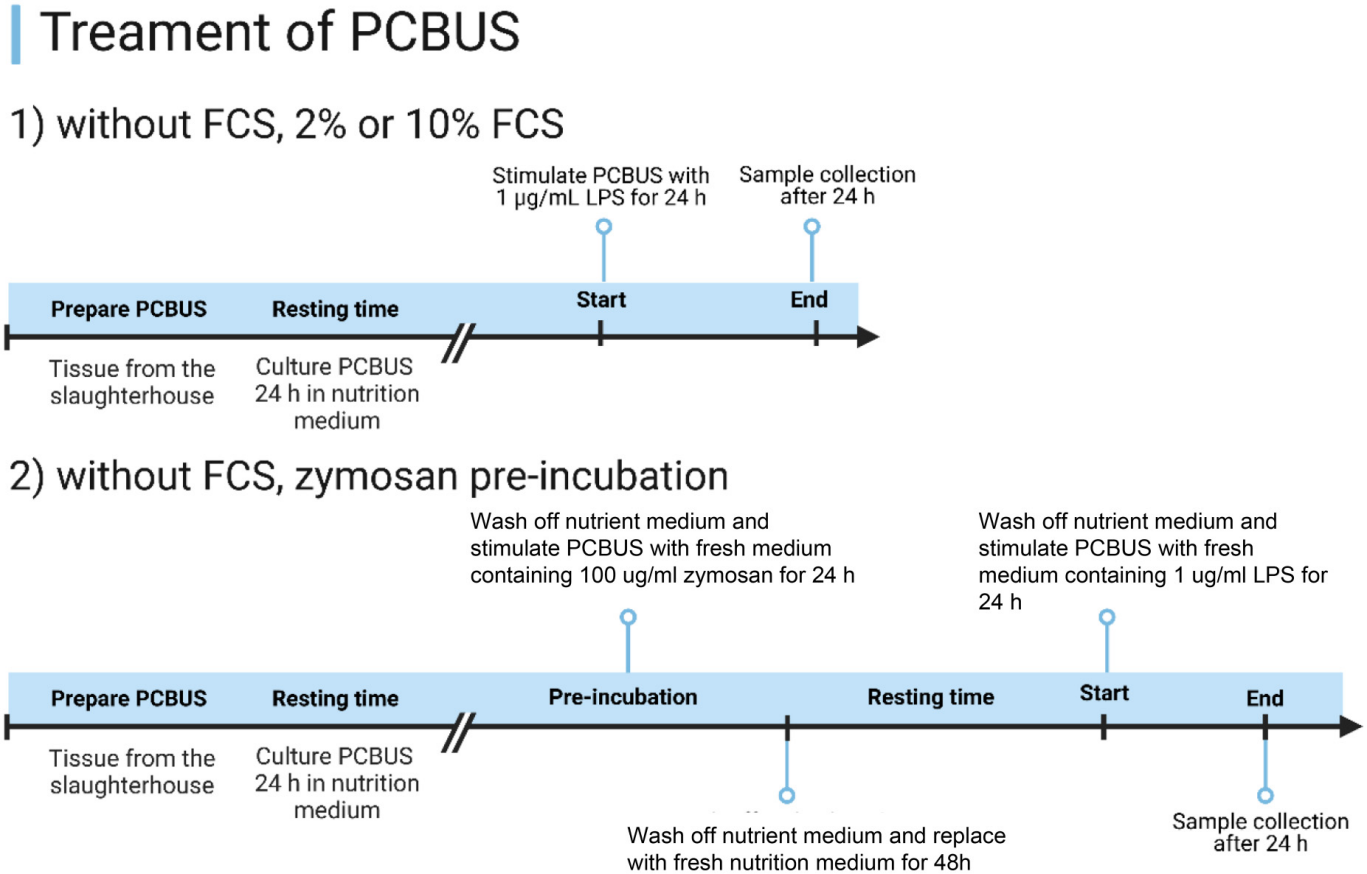
Experimental set-up of PCBUS stimulation.

First, investigating potential effects of FCS on cytokines release, we incubated slices under serum free, 2% FCS and 10% FCS conditions. In the second step, we then incubated the PCBUS with 1 µg/ml LPS from *E. coli* (O127:B8, Sigma) for 24 h. Control PCBUS were cultured with medium only. The supernatants of all groups were stored for enzyme-linked immunosorbent assay (ELISA) and multiplex analyses at −20°C. Furthermore, we were interested in the idea of trained immunity. Therefore, slices were cultured in serum free medium overnight before being incubated with 100 µg/ml zymosan (Sigma) for 24 h. Thereafter, slices were washed and the medium replaced. Slices were then left untreated for two days before being washed and stimulated with 1 µg/ml LPS for 24 h (Figure 1).

### Bovine cytokine/chemokine multiplex assay

Supernatants of PCBUS were analysed using a bovine customised multiplex assay kit (MILLIPLEX^®^ Bovine Cytokine/Chemokine Magnetic Bead Panel 1 - Immunology Multiplex Assay, Merck Millipore, UK). Cell culture supernatant samples were thawed, mixed and centrifuged at 1000 g for 10 min. Twenty-five µl of each sample were processed according to the manufacturer's instruction and analysed on the Bio-Plex 200 Systems (Bio-Rad, UK) to determine interferon (IFNγ), interleukin (IL)-1α, IL-1β, IL-4, IL-6, IL-8, IL-10, IL-17A, macrophage inflammatory protein (MIP)-1α, IL-36RA, interferon-inducible protein (IP)-10, monocyte chemoattractant protein (MCP)-1, MIP-1β, tumor necrosis factor (TNF)α and vascular endothelial growth factor (VEGF)-A. Bio-Plex Manager Software 6.2 was used to calculate concentrations from Median Fluorescent Intensity data. Only values ≥ lower limit of quantification (LLoQ; determined by the software) are shown in the graphs.

### Targeted lipid mediator profiling

Lipid mediators were identified and quantified using published protocols.^44^ Tissue samples from all udders were placed in ice-cold methanol containing deuterium labelled internal standards namely 500 pg of d_4_-LTB_4_, d_5_-MaR1, d_5_-MaR2, d_4_-PGE_2_, d_5_-LXA_4_, d_5_-RvD_3_, d_5_-RvD2, d_4_-RvE1, d_5_-d_5_-LTE_4_, d_5_-LTD_4_ and d_5_-LTC_4_ and 100 pg of 17R-RvD1, representing the chromatographic regions of interest. These were added to facilitate lipid mediator identification and quantification. Supernatants were extracted and lipid mediators were quantified using protocol described in ref.^44^ with minor modifications. Briefly, supernatants were subjected to solid-phase extraction using the ExtraHera system (Biotage) and Isolute C18 500 mg columns (Biogate). Methyl formate and methanol fractions were collected, brought to dryness, and suspended in phase (methanol/water, 1:1. vol/vol) for injection on a Shimadzu LC-20AD HPLC and a Shimadzu SIL-20AC autoinjector, paired with a QTrap 6500+ (Sciex). Analysis of mediators isolated in the methyl formate fraction was conducted as follows: an Agilent Poroshell 120 EC-C18 column (100 mm × 4.6 mm × 2.7 µm) was kept at 50 °C and mediators eluted using a mobile phase consisting of methanol/water/acetic acid of 20:80:0.01 (vol/vol/vol) that was ramped to 50:50:0.01 (vol/vol/vol) over 0.5 min and then to 80:20:0.01 (vol/vol/vol) from 2 min to 11 min, maintained till 14.5 min and then rapidly ramped to 98:2:0.01 (vol/vol/vol) for the next 0.1 min. This was subsequently maintained at 98:2:0.01 (vol/vol/vol) for 5.4 min, and the flow rate was maintained at 0.5 ml/min. In the analysis of mediators isolated in the methanol fraction, the initial mobile phase was methanol/water/acetic acid of 20:80:0.5 (vol/vol/vol) which was ramped to 55:45:0.5 (vol/vol/vol) over 0.2 min and then to 70:30:0.5 (vol/vol/vol) over 5 min and then ramped to 80:20:0.5 (vol/vol/vol) for the next 2 min. The mobile phase was maintained for 3 min and ramped to 98:2:0.5 (vol/vol/vol) for 2 min. QTrap 6500 + was operated using a multiple reaction monitoring method. Each lipid mediator was identified using the following criteria: (1) matching retention time to synthetic or authentic standards (±0.05 min), (2) signal/noise ratio ≥ 5 in a primary transition and (3) signal/noise ratio ≥ 3 in a secondary transition. Data was analyzed using Sciex OS v3.0, chromatograms were reviewed using the AutoPeak algorithm, using ‘low’ smoothing setting and signal to noise ratios were calculated using the relative noise algorithm. External calibration curves were used to quantify identified mediators. Where available calibration curves were obtained for each mediator using synthetic compound mixtures that gave linear calibration curves with R^2^ values of 0.98–0.99. These calibration curves were then used to calculate the abundance of each mediator per 1 ml of cell culture supernatant for each sample. Where synthetic standards were not available for the construction of calibration curves, calibration curves for mediators with similar physical properties (e.g., carbon chain length, number of double bonds, number of hydroxyl groups and similar elution time) were used.

### Biosynthetic pathway analysis

Differences between concentrations of lipid mediators from the groups were expressed as the Log_2_(fold change). Based on these differences, lipid mediator biosynthesis pathways were built using Cytoscape v.3.7.1.^45^ Pathways were made for each of the essential fatty acids and different lipid mediator family networks were illustrated using different line shapes. Up or downregulated mediators were denoted using upward and downward-facing triangles, respectively, and on changes in the node's size.

### Statistical analysis

All data were statistically analysed using GraphPad Prism 9.5.1 software (CA, USA). Unpaired t-test was applied to the obtained data. The trained immunity data were analysed using the Kruskal-Wallis test with a post-hoc Dunn's multiple comparison test to determine whether there was a significant difference in protein levels between all groups (control, Zym, Z + L and LPS). Data are presented as mean of three slices analysed per animal, and graphs were generated using R software (version 4.4.2). *P* < 0.05 was set as the significance level.

Principal Component Analysis (PCA) and Partial least squares-discrimination analysis (PLS-DA) were performed using MetaboAnalyst v5^46,47^ after mean centering and unit variance scaling of lipid mediator concentrations. The score plots illustrate the clustering among the different samples (closest dots representing higher similarity). The scree plot displays the contribution of each of the principal components to the total variation in a dataset. The green line shows the cumulative variance explained by the first five components, while the red line displays the individual variance for each principal component.

## Results

### Viability assay and morphological analysis

**Figure 2.**
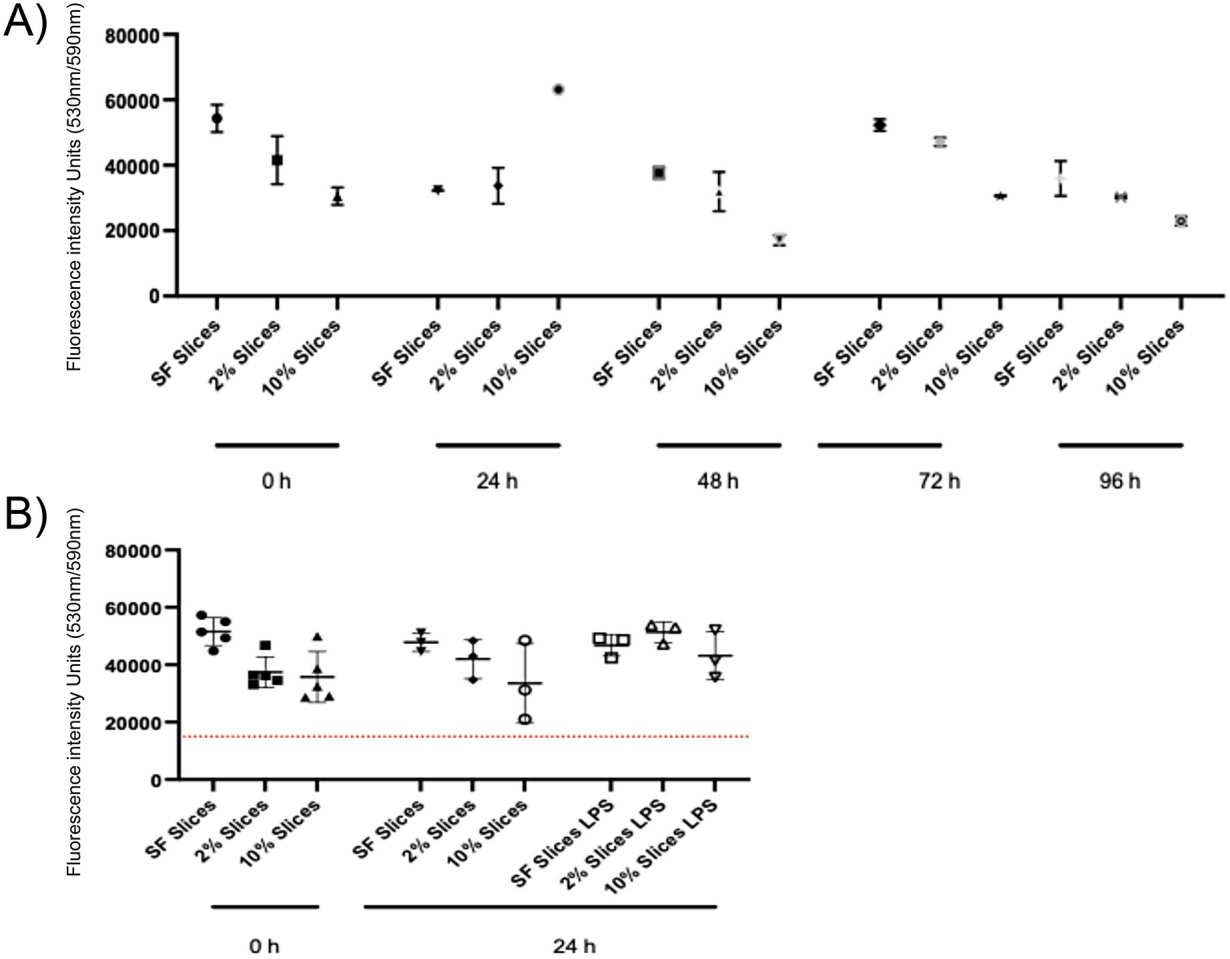
(A) Alamarblue survival assay for (PCBUS) in different concentration of FCS over a five day period. Values are shown as mean of 3 slices generated from 3 different animals. Slices were kept in indicated media for up to 96 h, before survival was measured using AlamarBlue assay as described. (B) AlamarBlue survival assay for (PCBUS) in different concentration of FCS and left untreated or stimulated with 1 µg/ml LPS. Values are shown as mean of 3 slices generated from 3 different animals. Slices were kept in indicated media for up to 24 h, before survival was measured using AlamarBlue assay as described.

**Figure 3.**
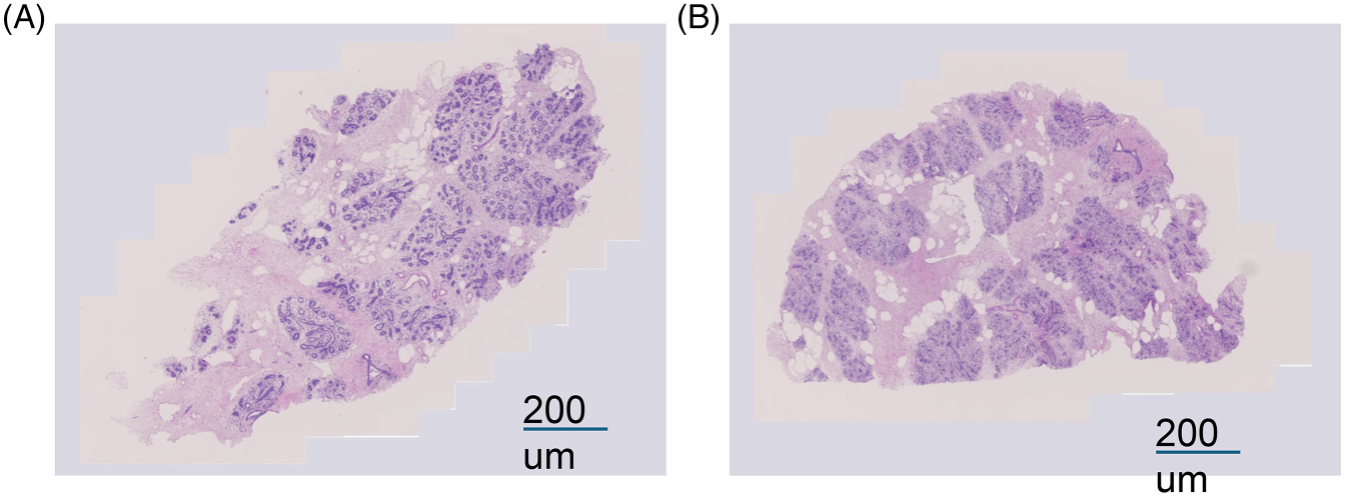
(A) Hematoxylin-eosin staining of (PCBUS) in serum free medium right after processing showing physiological morphology of bovine udder; (B) hematoxylin-eosin staining of PCBUS in serum free medium 5 days after processing showing physiological morphology of bovine udder. Magnification 20× for both micrographs. Slides were stained for H&E using an autostainer, and subsequently were scanned using an AxioScan Z1 slidescanner (Serial number: 4647000114 C) with Zen Blue (Version 3.1.) software package.

The viability of the PCBUS was assessed every 24 h and showed that they were viable throughout the study period (Figure 2A and B). Histological images taken of slices directly after cutting showed that a typical udder morphology was maintained after PCBUS processing. In contrast, after 5 d in culture, lumen of milk ducts were less clear visible, but the epithelial barrier was still intact (Figure 3A and B).

### FCS affects the release of immune mediators in different ways

**Figure 4.**
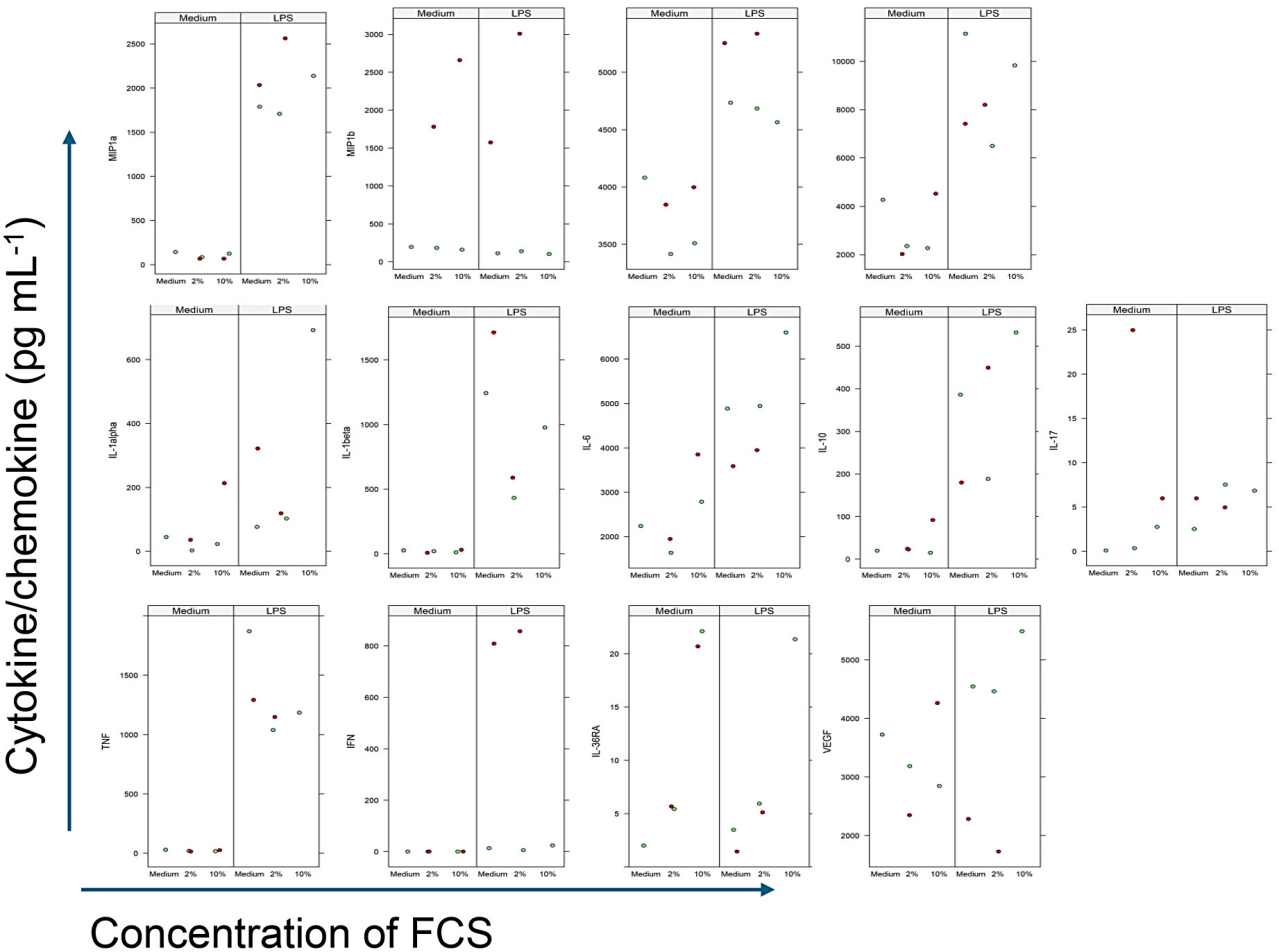
Influence of FCS on the induction of immunomodulator profile of precision-cut bovine udder slices (PCBUS) left untreated or stimulated with 1 µg/ml LPS. Analysis of immunomodulators was performed in supernatants of PCBUS generated from 3 different udders, each analyzed in triplicate, by multiplex assay. Data are presented as mean of the three technical repeats per udder. Figure 4A depict values obtained for chemokines, Figure 4B those obtained for cytokines, Figure 4C for remaining immunomodulators. Identical colours depict slices generated from the same animal.

As there is an on-going discussion regarding the use of fetal calf serum (FCS) on phenotype, morphology and functionality of cells,^48,49^ we investigated in the first set of experiments the impact of different concentrations of FCS on cytokine production of LPS-stimulated PCBUS by multiplex assay. The results for immunomodulators with significant differences are shown in Figure 4.

While stimulation of PCBUS with LPS had an effect on the concentration of chemokines analysed, different concentrations of FCS did not impact consistently on chemokine production (Figure 3A). For all chemokines determined by the multiplex assay, there was a clear induction by LPS (Figure 4A).

The response pattern varied with regards to the cytokines analysed. In general, LPS-stimulation of PCBUS resulted in an increase in cytokine production (Figure 4B). However, with regards to the addition of FCS, three different response patterns could be observed. For one group of cytokines, including IL-1α, IL-6, IL-10 and IL-36RA, the response to LPS was greater with increasing concentrations of FCS to various degrees (Figure 4B). In contrast, the production of another group of cytokines, including IL-1β, TNFα and IL-17A, decreased with increasing concentrations of FCS (Figure 4B and C). For a third group, including IFNγ and VEGF, the incubation of PCBUS in different concentrations of FCS had no apparent effect on LPS-induced cytokine production (IFNγ and VEGF, Figure 4B and C).

It is interesting to note that IL-1β and IL17A are cytokines shown previously in *in vivo* experiments to play a substantial role in E.coli mastitis.^50,51^ These two cytokines are either leading to a stimulation of a local as well as systemic response (in case of IL-1β), while IL-17A is not only a mucosal defence cytokine,

### Zymosan significantly modulates the immune response to LPS

Due to the different influences of FCS on the release of interleukins by LPS-stimulated PCBUS, but especially the effect of FCS on the control group, all further experiments assessing potential trained immunity in response to zymosan (zym) and LPS were performed in serum-free medium.

**Figure 5.**
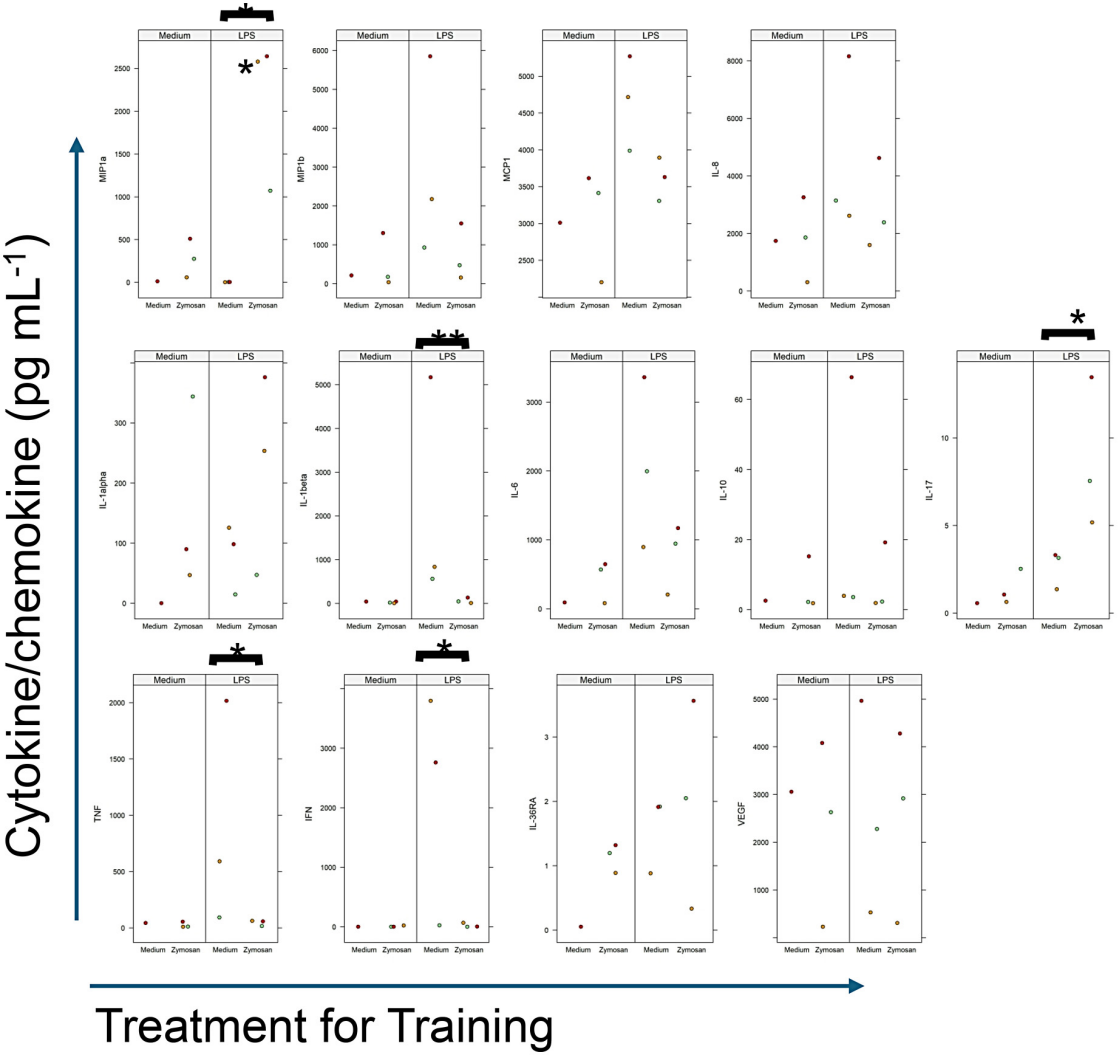
Influence of trained immunity on the chemokine/cytokine profile after stimulation of precision-cut bovine udder slices (PCBUS) with LPS stimulation alone (24 h). PCBUS were treated as described, and supernatants of PCBUS generated from 3 different udders, each analyzed in triplicate, were assayed by multiplex assay. Data are presented as mean of the three technical repeats per udder (*p ≤ 0.05; **p ≤ 0.01). Figure 5A depict values obtained for chemokines, Figure 5B those obtained for cytokines, Figure 5C for remaining immunomodulators. Only values above the  lower limit of quantification (LLoQ; determined by the software) are shown in the graphs. Identical colours depict slices generated from the same animal.

For the majority of immunomodulators tested, training with zymosan resulted in a higher production when PCBUS were incubated without LPS (Figure 5). With regards to the LPS response after training, the reaction can be grouped in two main reactions: on one side, zymosan training increased the production of MIP-1α (Figure 5A), IL-1α (Figure 5B), potentially IL-36RA (Figure 5C), but specifically IL-17A (Figure 4B). In contrast, training reduced the response of all other immunomoduators to stimulation with LPS, notable IL-1β (Figure 5B), IFNγ (Figure 5B) and TNFα (Figure 5C).

### Modulation of eicosanoids and specialised pro-resolving lipid mediators

Lipid mediators play a crucial role in both the initiation and resolution of inflammation. Consequently, we evaluated whether lipid mediators are regulated in PCBUS in response to zymosan and/or LPS stimulation. Utilising established methodologies, we identified mediators from the four essential fatty acid metabolomes within these tissue slices. These include resolvins derived from docosahexaenoic acid (DHA), n-3 docosapentaenoic acid (n-3 DPA), and eicosapentaenoic acid, along with DHA-derived maresins, and arachidonic acid (AA)-derived lipoxins, leukotrienes, and prostaglandins (Table 1).

**Table 1.** Lipid mediator profiles identified in PCBUS.

	MRM transitions			Control			Zymosan			LPS			Zymosan + LPS		
DHA-derived mediators	Q1	Q3 (Quantifier)	Q3 (Qualifier)	Mean	±	SEM	Mean	±	SEM	Mean	±	SEM	Mean	±	SEM
RvD1	375	141	215	0.01	±	0.00	0.03	±	0.03	0.02	±	0.01	0.02	±	0.01
17R-RvD1	375	215	-		*			*			*			*	
RvD2	375	215	-		*			*			*			*	
RvD3	375	137	-		*			*			*			*	
17R-RvD3	375	137	147	0.02	±	0.02	0.16	±	0.16	0.11	±	0.11	0.04	±	0.04
RvD4	375	101	-		*			*			*			*	
RvD5	359	199	225		*			*			*			*	
RvD6	359	159	101		*			*			*			*	
PD1	359	137	153	0.36	±	0.36		*			*			*	
PDX	359	153	123	0.53	±	0.49	0.05	±	0.04	0.02	±	0.01	0.02	±	0.01
17R-PD1	359	123	-		*			*			*			*	
PCTR1	650	308	325	0.16	±	0.12	0.48	±	0.30	0.23	±	0.05	0.22	±	0.17
PCTR2	521	213	-		*			*			*			*	
PCTR3	464	245	-		*			*		0.06	±	0.03	0.10	±	0.05
MaR1	359	221	-		*			*			*			*	
MaR2	359	191	221	0.06	±	0.02	0.10	±	0.09	0.00	±	0.00	0.02	±	0.02
MCTR1	650	308	191	0.16	±	0.12	0.35	±	0.16	0.19	±	0.10	0.19	±	0.19
MCTR2	521	191	-		*			*			*			*	
MCTR3	464	173	191	0.13	±	0.06	0.55	±	0.47	0.25	±	0.11	0.22	±	0.12
*n-3 DPA-derived mediators*															
RvT1	377	211	193		*			*			*			*	
RvT2	377	143	209		*		0.01	±	0.01	0.01	±	0.01	0.01	±	0.01
RvT3	377	215	-		*		0.15	±	0.15		*			*	
RvT4	361	211	143	0.13	±	0.13		*		0.05	±	0.05	0.12	±	0.05
RvD1n-3 DPA	377	143	-		*			*			*			*	
RvD5n-3 DPA	361	143	199	0.13	±	0.08	0.12	±	0.05	0.09	±	0.04	0.11	±	0.05
PD1n-3 DPA	361	263	155	0.16	±	0.16		*			*			*	
*EPA-derived mediators*															
RvE1	349	161	-		*			*			*			*	
RvE2	333	253	159	0.04	±	0.04	0.06	±	0.06	0.03	±	0.01	0.05	±	0.04
RvE3	333	201	253	2.13	±	1.13	1.23	±	1.23	0.75	±	0.75	*	*	*
RvE4	333	253	115	0.01	±	0.01	0.02	±	0.02	*	*	*	0.06	±	0.05
*AA-derived mediators*															
LXA_4_	351	235	-		*			*			*			*	
LXB_4_	351	233	163	27.01	±	16.36	54.73	±	32.77	62.68	±	31.87	99.94	±	15.80
5S,15S-diHETE	335	201	115	0.49	±	0.28	0.38	±	0.23	0.09	±	0.02	0.11	±	0.02
15-epi-LXA_4_	351	115	-		*			*			*			*	
LTB4	335	195	-		*			*			*			*	
20-OH-LTB4	351	195	-		*			*			*			*	
LTC_4_	626	189	301	0.01	±	0.01	0.01	±	0.01	0.01	±	0.01		*	
LTD_4_	497	301	189	0.02	±	0.02	0.03	±	0.03		*		0.09	±	0.09
LTE_4_	440	301	189	0.02	±	0.02	0.01	±	0.01	0.02	±	0.02	0.09	±	0.04
PGD_2_	351	189	-		*			*			*			*	
PGE_2_	351	189	271	38.88	±	24.35	86.96	±	51.84	117.55	±	55.29	239.10	±	74.14
PGF_2a_	353	247	193	0.74	±	0.26	3.88	±	1.88	4.81	±	2.22	16.55	±	10.85
TxB_2_	369	195	169	0.91	±	0.24	1.84	±	0.35	3.73	±	1.96	25.43	±	11.59

Lipid mediators were extracted using C18 SPE and identified and quantified in PCBUS using LC-MS/MS based methodologies. * = Below lower limits of quantitation.

MRM: multiple reaction monitoring; SEM: standard error of mean.

**Figure 6.**
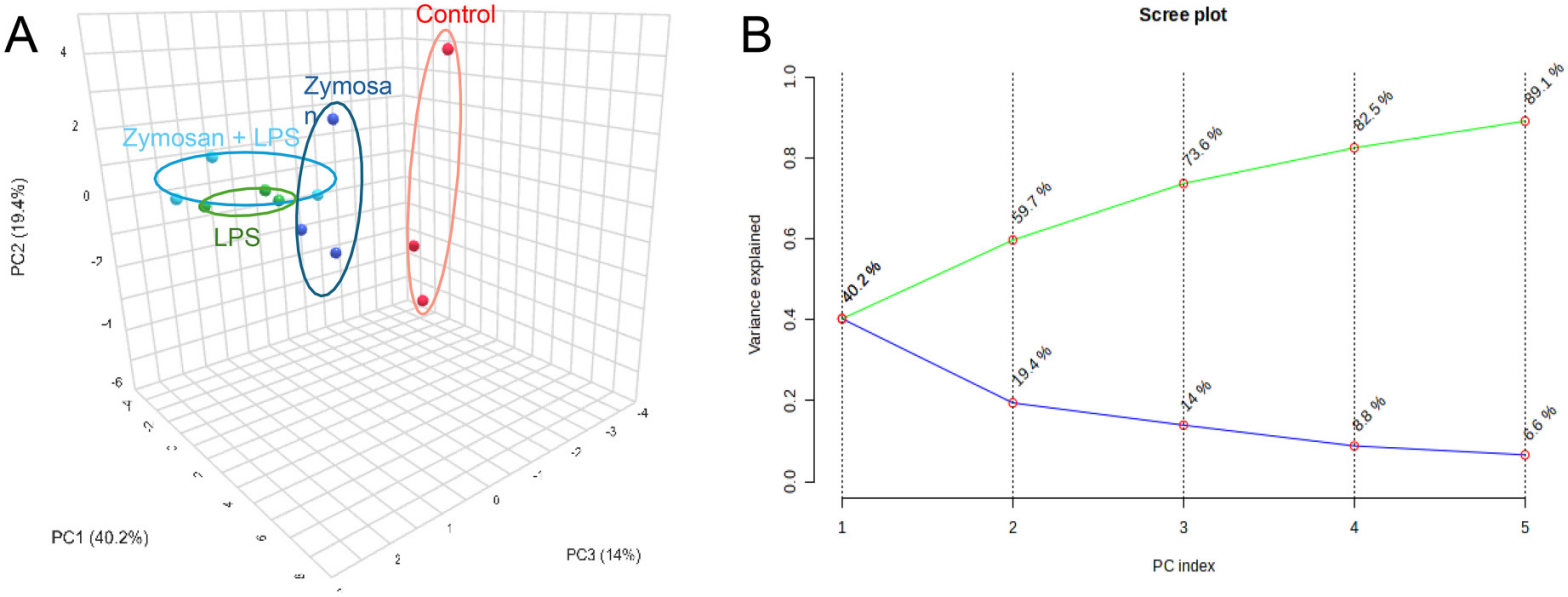
Regulation of lipid mediator profiles in PCBUS after induction of trained immunity and subsequent stimulation by LPS. Lipid mediators were extracted using C18 SPE and lipid mediators were identified and quantified using LC-MS/MS. Regulation of lipid mediator levels was evaluated using principal component analysis (A) Scores Plot (B) Scree Plot.

We then used principal component analysis to assess the relative regulation of these mediators across different experimental groups. The analysis revealed that incubation with zymosan and/or LPS induced a significant shift in overall lipid mediator levels, as evidenced by the clustering pattern of tissues treated with zymosan and/or LPS compared to the control groups. The most pronounced differences were observed in PCBUS incubated with either LPS alone or after induction of trained immunity and subsequent stimulation with LPS (Figure 6A and B).

Further evaluation of individual lipid mediator concentrations showed that pro-inflammatory stimuli led to an increase in AA-derived specialised pro-resolving mediators, such as LXB_4_, and pro-inflammatory eicosanoids, including prostaglandin (PG)E_2_ and thromboxane (TX)B_2_ the breakdown product of the pro-thrombotic mediator TXA_2_. Additionally, we noted a decrease in DHA-derived SPMs like MaR2 (as shown in Table 1), although these changes were not statistically significant. Collectively, these findings highlight the presence and regulation of lipid mediators in PCBUS following zymosan and/or LPS incubation.

## Discussion

The development of antimicrobial resistance (AMR) is a major challenge for global health, closely related to the excessive use of antibiotics in cattle, with prominence for bovine mastitis. Indeed, at any given time point, roughly 20% of cows suffer from mastitis and require antibiotic treatment.^52^ Given that antibiotic usage has to be reduced, different treatment protocols are needed to address this problem in practice and to explore new non-antibiotic alternatives for prevention and treatment of mastitis. One of be biggest problems with regards to mastitis is the lack of a protective immune memory generated by either undergone disease or current vaccines. While immune memory is considered a defining feature of the acquired immune system, the activation of the innate immune system can also result in enhanced responsiveness to subsequent triggers. This process has been termed ‘trained immunity’, a de facto innate immune memory, and is mainly driven by macrophages. Interestingly, a set of macrophages associated with the mammary gland and lactation were recently identified in the mammary gland in mice and humans.^53^ These cells developed independently of IL-34, but required CSF-1 signalling and were partly microbiota-dependent. Locally, they resided adjacent to the basal cells of the alveoli and extravasated into milk. Collectively, these findings reveal the emergence of unique macrophages in the mammary gland milk during lactation that could contribute to an innate immune memory. However, trained immunity is only short lived (weeks to months), and is still difficult to assess both, *in vitro* and *in vivo*. In the first instance, we assessed the impact of FCS on the use of PCBUS as a model to assess whether trained immunity can be induced in PCBUS. The standardised addition of FCS can strongly influence the immune response of the cells, as there is a high product variability in the composition of the individual components.^41,54^ In addition, pathogens have been detected over the years despite filtration techniques.^42,55^ With this in mind, it was important for us to first investigate whether PCBUS could be cultured without the addition of FCS to assess whether the release of immune mediators is due to the stimulus added or a by-product of exposure to FCS.

Despite the fact that we used PCBUS from different animals, we were somewhat surprised to see an inconsistent effect of FCS on cytokine production/release. While the concentration of IL-1ß decreased with the addition of FCS, a slight increase in the concentration of IL-6 with increasing FCS supplementation in the LPS-stimulated group was observed. There was an almost twofold increase in IL-6 levels in the medium of the SF control group if supplemented with 10% FCS. This suggests that FCS may stimulate the release of IL-6 from cells within the PCBUS. A similar effect was observed for IL-17a. Although the mean value of the control groups varied widely, the number of PCBUS that appeared to secrete IL-17a was doubled by the addition of FCS alone.

Since the PCBUS remain viable and morphologically and physiological unaltered throughout the experiment without the addition of FCS, all subsequent experiments were performed using serum-free condition to assess the impact of pre-incubation with zymosan and its ability to induce trained immunity in PCBUS.

The cytokine profile for IL-1ß shows that pre-incubation of PCBUS with zymosan followed by stimulation with LPS did not result in secretion of IL-1ß. However, LPS alone was able to induce a significant increase compared to all three groups. Based on the literature in the field of trained immunity, we expected an increased secretion of IL-1ß in the Z + L group compared to the LPS group.^13^ Our results suggest that zymosan, similar as described for LPS,^56^ may induce an endotoxin tolerance. IL-6 levels were also highest in the LPS-stimulated group. Again, pre-incubation with zymosan did reduce the IL-6 secretion in response to LPS. Only IL-17A was found to be elevated in the Z + L group. The literature suggests that trained immunity can lead to long-term effects, especially in T-cell responses.^57^ This raises the question whether the pretreatment with zymosan could be optimised by using different concentrations and incubation times to induce quicker and stronger cytokine/chemokine responses in the presence of LPS and avoid tolerance. Indeed, the optimisation of the stimulus to recall innate memory could further improve the cytokine response. For further investigation, it would also be interesting to clarify which cell types and receptors can be detected in the PCBUS, such as tissue-resident macrophages, dectin-1 and TLR receptors.

In addition to the cytokine response, we also analysed lipid mediators, resulting in a treatment-specific response when analysed by principal component analysis. We noticed that pro-inflammatory lipd mediators and lipid derivates, such as, PGD_2_, PGE_2_, PGF_2a_ and TXB_2_ (the non-enzymatic breakdown product of the pro-thrombotic mediator TXA_2_) were upregulated in samples that were pre-treated with zymosan before LPS stimulation. The fact that LPS induces these prostaglandins *in vivo* has been documented before.^58^ Prostaglandins are lipid-derived autacoids formed from arachidonic acid. They play a dual role in maintaining cellular and tissue homeostasis, but also drive pathological processes, such as inflammation. Their production is catalysed by cyclooxygenase (COX) enzymes, and their synthesis can be inhibited by nonsteroidal anti-inflammatory drugs (NSAIDs), including COX-2 selective inhibitors. This resulted in the notion that the use of NSAIDS could potentially be enough to treat Gram-negative bacteria causing mastistism replacing the classical antibiotic therapy approach for acute mastitis forms (reviewed in ref.^59^). It is believed that under such conditions the damage to tissue may not solemnly be based on the infection, but is enhanced by an overshooting immune response. “Resetting” this response through NSAIDS may therefore reduce this response, giving the host the ability to clear the pathogen more effectively, resulting in a faster restoration of the tissue. While NSAIDs are clinically effective, prostaglandins are involved in both initiating and resolving inflammatory responses. Prostaglandins and TXA_2_, collectively known as prostanoids, are derived from arachidonic acid (AA), a 20-carbon unsaturated fatty acid. Their synthesis begins with the release of AA from the plasma membrane by phospholipases (PLAs), followed by its metabolism through the sequential actions of prostaglandin G/H synthase (commonly referred to as cyclooxygenase, or COX) and specific synthases. This results in the production of four primary bioactive prostaglandins: PGE_2_, PGI_2_, PGD_2_, and PGF_2α_. These are widely synthesised, with most cell types producing one or two dominant types, and they function as autocrine and paracrine lipid mediators to regulate local homeostasis. During inflammation, the levels and types of prostaglandins changes significantly. In healthy, uninflamed tissues, prostaglandin production is typically relatively low. However, during acute inflammation, their production rapidly increases, preceding the recruitment of leukocytes and infiltration of immune cells. Intriguingly this increase in pro-inflammatory eicosanoids was linked with an overall deacrease in pro-resolving mediators supporting the emergence of an overall pro-inflammatory lipid mediator profile. Together, we believe that PCBUS clearly show the expected responses, and that zymosan is indeed inducing a “trained immunity” mechanism, similar as described for BCG which was found to upregulate prostaglandin production in monocytes.^60^

Unfortunately, while the current data are encouraging, we cannot draw yet a clear conclusions regarding the concept of trained immunity in PCBUS in our current study. While the analysis of some mediators, such as prostaglandins and IL-17A clearly showed a “trained immunity” response, this was not seen for other known cytokines. Future studies need to critically re-evaluate the experimental design, in particular the concentration of zymosan, the timing and length of pre-incubation, and the use of zymosan as an inducer of trained immunity. Our results of the studies on the use of FCS indicate, that there is no need to add this complex, inhomogeneous supplement for successful PCBUS cultivation. Indeed, standardisation of experiments not using FCS would potentially make these more comparable across different laboratories, increasing reproducibility of data. Furthermore, the absence of FCS in these experiments, and maybe others, reduce the need for fetal calves, thus being in line with 3R guidelines. Indeed, we have shown that PCBUS can remain viable for more than 24 h even when stimulated with LPS without the addition of FCS and can respond to external stimuli by secreting immune mediators. This is an important step towards animal-free or at least animal-need reduced research in the field of immunology and pharmacology, but raises further questions whether FCS may be a good additive in other *in vitro* settings, too.
